# Serum oxidative stress in patients with pulmonary *Mycobacterium avium* complex disease

**DOI:** 10.1016/j.heliyon.2019.e02775

**Published:** 2019-11-14

**Authors:** Hiroki Wakabayashi, Yasuo Matsuzawa, Sho Hayakawa, Tamako Irie, Hagino Rikitake, Ichiro Tatsuno

**Affiliations:** a Department of Internal Medicine, Toho University Sakura Medical Center, Japan; b Department of Diabetes, Endocrinology and Metabolism, Toho University Graduate School of Medicine, Japan

**Keywords:** Microbiology, Infectious disease, Internal medicine, Medical imaging, Respiratory system, Biomarkers

## Abstract

**Background:**

The mechanism of progressive airway destruction in incurable chronic infection of the lung – termed pulmonary *Mycobacterium avium* complex (pMAC) disease – is currently unknown. The involvement of oxidative stress in a variety of progressive chronic respiratory diseases has been previously reported. It has been hypothesized that oxidative stress may be involved in the progression of airway destruction in pMAC disease.

**Patients and methods:**

The study included 28 untreated patients with pMAC disease. The level of serum oxidative stress was quantitatively evaluated through the diacron reactive oxygen metabolites (d-ROMs) test, which indirectly measures the level of hydroperoxide in the serum. In addition, patients were divided into three groups based on the severity shown in the computed tomographic image.

**Results:**

The level of serum oxidative stress exceeded the normal range (250–300 U.Carr [Carratelli Units]) in all patients with pMAC disease (mean: 495.5 ± 102.6 U.Carr; minimum–maximum: 340–734 U.Carr). The level of serum oxidative stress in patients with severe disease was significantly higher compared with that observed in patients with mild disease (434.6 ± 30.2 vs. 583.4 ± 95.1, respectively, p = 0.009).

**Conclusions:**

In patients with pMAC disease, an elevation was observed in the level of serum oxidative stress. This increase in oxidative stress was more pronounced in patients with severe disease.

## 1 Introduction

Nontuberculous mycobacteria (NTM) are indigenous bacteria present in water and soil. Although these organisms usually do not show pathogenicity, they can persistently infect the lungs of certain hosts and cause pulmonary NTM (pNTM) disease. The progression of pNTM lesions varies among individual hosts. Notably, the disease may not progress even in the absence of pharmacological treatment, or may progress and become fatal regardless of treatment. Among NTMs, the *Mycobacterium avium* complex (MAC) accounts for 90% of the causative bacteria in Japan [1]. In addition, pulmonary MAC (pMAC) disease is the most frequent infectious disease among pNTMs [2, 3].

Unlike tuberculosis, MAC involves indigenous bacteria and does not show pathogenicity in numerous individuals. Nevertheless, it may cause opportunistic infections in patients with immunodeficiency (e.g., acquired immunodeficiency syndrome) [4]. On the other hand, MAC occasionally infects "healthy individuals" without immune deficiency and causes lung lesions. Currently, the standard treatment of pMAC disease involves combination therapy with clarithromycin, ethambutol, rifampicin, etc. However, despite the administration of this standard regimen, airway destruction (i.e., bronchiectasis and cavity lesions) often progresses, leading to death due to respiratory failure [3, 5]. The mechanism through which MAC infects healthy individuals, resulting in destruction of the bronchia and lungs despite treatment with antibiotics, remains unknown.

It has been reported that oxidative stress is involved in various pulmonary inflammatory diseases, leading to structural destruction of the airways and lungs [6, 7, 8, 9]. The relationship of pulmonary emphysema and pulmonary fibrosis with oxidative stress has been widely studied. Evidence has shown that serum oxidative stress increased in bronchiectasis [7]. Further studies reported that serum oxidative stress was also elevated in sarcoidosis, resulting in fibrosis due to chronic granulomatous inflammation [9]. However, the association between pMAC disease and oxidative stress remains poorly understood.

The diacron reactive oxygen metabolites (d-ROMs) test is a method indirectly measuring the level of oxides (i.e., hydroperoxides). Hydroperoxides are metabolites produced by active oxygen and free radicals following the oxidation of lipids, proteins, amino acids, nucleic acids, etc. that constitute the body. It is thought that oxidative stress reflects the production of cytokines by the inflammatory cells of the host due to invasion, and that the d-ROMs test is able to quantitatively evaluate this reaction. We previously reported that changes in the level of serum oxidative stress – measured using the d-ROMs test – were associated with progression of pulmonary fibrosis in idiopathic pulmonary fibrosis (IPF) patients [10].

We hypothesized that oxidative stress also plays a role in the progressive mechanisms of bronchodilation and lung cavitation in patients with pMAC disease. To test this hypothesis, we measured the level of oxidative stress in patients with pMAC disease through the d-ROMs test. Moreover, we examined the association between this level and the severity of disease.

## 2 Materials & methods

### 2.1 Patients and study design

This retrospective study included 28 patients with pMAC disease who visited our outpatient division at the Department of Respiratory Internal Medicine, Toho University Medical Center Sakura Hospital, Sakura, Japan from April 2011 to March 2016. The disease (pMAC) was diagnosed according to the 2007 American Thoracic Society/Infectious Disease Society of America (ATS/IDSA) guidelines [4] and the following three criteria: (1) different sputum cultures were MAC-positive or bronchoalveolar lavage fluids were MAC-positive at least once; (2) computed tomography (CT) and diagnosis of pMAC disease were performed by a least one pulmonologist and at least one radiologist; and (3) lung diseases other than pMAC disease (i.e., lung cancer, interstitial pneumonia, etc.) were excluded. In order to exclude the variation of oxidative stress associated with the use of antibiotics, only patients who had not received combination antibiotic therapy for pMAC disease within the previous 2 years were included in the analysis. Furthermore, patients with colonization by other microorganisms observed in sputum culture, and those who exhibited symptoms of acute respiratory infection in the previous 8 weeks were excluded. The body mass index, total protein, albumin, aspartate aminotransferase, alanine aminotransferase, lactate dehydrogenase, alkaline phosphatase, c-reactive protein, and white blood cell count were measured to evaluate the clinical background of the patients. This study was approved by the Ethics Committee of Toho University on November 30, 2016 (Ethics Committee of Toho University). All patients included in this study provided written informed consent.

### 2.2 d-ROMs test

Oxidative stress was evaluated through measurement of serum hydroperoxide using the d-ROMs test. The Free Radical Analytical System 4™ (Wismerll Co. Ltd., Tokyo, Japan) was used for the test. The principle of the test is based on the concept that the content of organic hydroperoxide in the serum reflects that of free radicals responsible for its production. The test procedures were performed as follows. Blood samples collected from a peripheral vein of the patients were centrifuged at 4 °C at 1,500×g for 15 min. Subsequently, 20 μl of serum were mixed with acid buffer solution (pH 4.8) in a cuvette, and supplemented with 20 μl of the chromogen. Serum hydroperoxide reacts with the transition metal ion released from the protein in the blood under acidic conditions, changing to alkoxy or peroxy radicals. These newly produced radicals oxidize the chromogen, yielding a purple product.

The concentration of the stable product was measured using a spectrophotometer (absorbance at 505 nm). The normal range of the test results was 250–300 U.Carr (Carratelli Units). Notably, 1 U.Carr corresponds to 0.8 mg/l of hydrogen peroxide. Values exceeding 300 U.Carr indicated increased oxidative stress.

### 2.3 Chest radiographic and CT findings

All patients underwent CT. The patients were classified into the following three groups according to the spread of their lesion: 1) mild, lesion area confined to one lobe of one lung; 2) severe, lesion area extending beyond one lung; and 3) moderate, lesion area between mild and severe.

### 2.4 Statistical analysis

In cases without annotation, data are expressed as mean ± standard deviation. Student's *t*-test was used for all comparisons between two independent groups. An overall difference between the groups was determined using one-way analysis of variance. When the results of this analysis were significant, differences between individual groups were determined using Fisher's test.

Correlations between variables were analyzed using the Pearson correlation coefficient and Spearman's rank correlation coefficient. Multiple linear regression analysis was used to clarify the effects of confounding factors on the level of oxidative stress. This analysis was performed by implementing correlation analyses for each parameter, the level of oxidative stress, and the severity grading based on the CT images. This was followed by the use of a variable increasing method, which adds variables in an ascending manner (i.e., starting from those with the lowest *p* value in the univariate analysis). All statistical analyses were performed using the SPSS 21.0 software (IBM CO., Armonk, NY, USA). A *p* < 0.05 denoted statistical significance.

## 3 Results

**Fig. 1:**
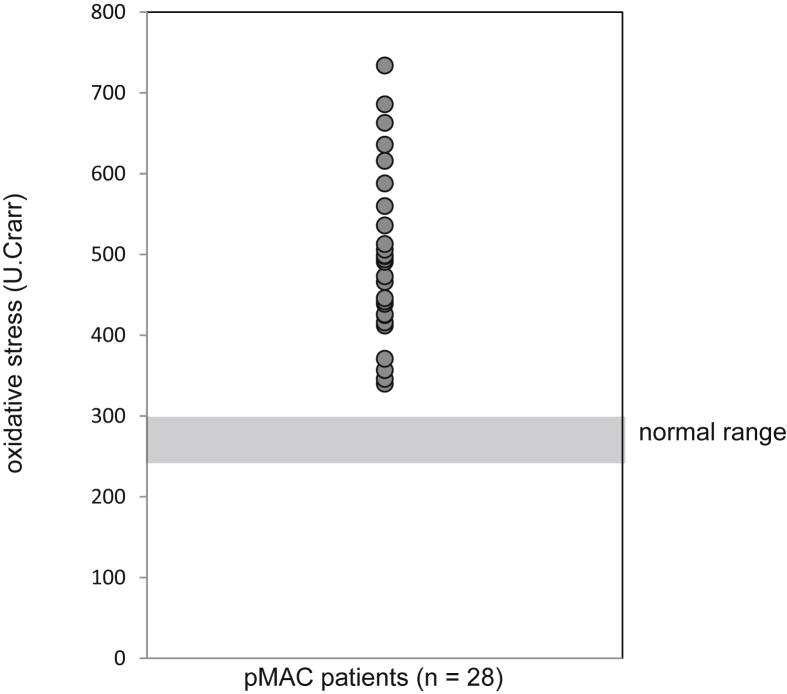
These levels exceeded the upper limit of the normal reference value. pMAC: pulmonary *Mycobacterium avium* complex; U.Carr: Carratelli Units.

**Fig. 2:**
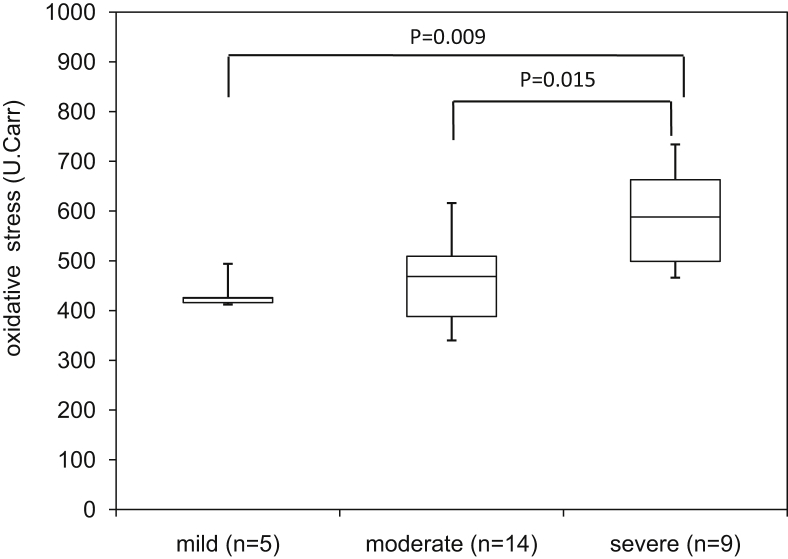
The level of serum oxidative stress in the severe group was significantly higher than those reported in the mild and moderate groups.

The background factors of the 28 patients with pMAC disease included in this study are shown in Table 1. The mean level of serum oxidative stress was 495.5 ± 102.6 U.Carr. In all patients, the levels exceeded the upper limit of the normal reference value (i.e., 300 U.Carr) (Fig. 1). According to the CT-grade (i.e., severity grading based on the CT images), 5, 14, and 9 patients were classified into the mild, moderate, and severe groups, respectively (Table 2). The corresponding d-ROMs test values were 434.6 ± 33.7, 460.6 ± 84.7, and 583.4 ± 100.9 U.Carr, respectively (Fig. 2). Significant differences were observed in the mean level of serum oxidative stress among these three groups *(p* = 0.003). Notably, the level of serum oxidative stress in the severe group was significantly higher compared with those measured in the mild and moderate groups (*p* = 0.009).

**Table 1:** Data are presented as mean ± SD, unless otherwise indicated.

	pMAC patients	Normal value
N	28	
Age mean±SD	65.2±11.8	
Sex (m/f)	5/23	
BMI (kg/m2) mean±SD	18.6±2.7	
d-ROMs (U,CARR) mean±SD	495.5±100.5	250–300
T-P (g/dl) mean±SD	7.8±0.6	6.7–8.3
Alb (g/dl) mean±SD	4.4±0.4	3.8–5.2
AST (IU/l/) mean±SD	22±5	10–40
ALT (IU/l/) mean±SD	15±6	5–45
LDH (IU/l/) mean±SD	187±26	120–240
ALP (IU/l/) mean±SD	254±71.7	100–325
CRP (mg/dl) mean±SD	1.1±2.4	≦0.30
WBC (/μl) mean±SD	6699±2435	3300–9000
CT grade (mild/moderate/severe)	5/14/9	

BMI: body mass index, d-ROMs: diacron reactive oxygen metabolites, T-P: total protein, Alb: albumin, AST: aspartate aminotransferase, ALT: alanine aminotransferase, LDH: lactate dehydrogenase, CRP: C-reactive protein, WBC: white blood cell count, CT: computed tomography; SD: standard deviation.

**Table 2:** Patients with pMAC disease were divided into three groups based on the CT-grade (i.e., severity grading based on the CT images).

CT grade	Mild	Moderate	severe
N	5	14	9
Age (yr) mean±SD	55.2±17.9	64.9±7.47	71.3±8.54
Sex (m/f)	1/4	2/12	2/7
BMI (kg/m^2^) mean±SD	18.1±2.2	18.7±2.4	18.7±3.3
d-ROMs (U,Carr) mean±SD	434.6±30.2	461±81.7	583.4±95.1

pMAC: pulmonary *Mycobacterium avium* complex; CT: computed tomography.

Multiple classification analysis was performed to examine the effects of confounding factors (other than CT-grade), on the level of serum oxidative stress. The patients were categorized into two groups for this analysis according to the CT-grade: a mild/moderate group and a severe group (two-step CT-grade). The correlation coefficients between the level of oxidative stress and each parameter are shown in Table 1, while the oxidative stress values are shown in Table 3. Among them, the parameters which indicated a significant correlation were selected, and a multiple regression analysis was performed (Table 4). Only two-step CT-grade showed a significant correlation with serum oxidative stress (*p* = 0.043), and was considered an independent predictor of oxidative stress.

**Table 3:** Correlation coefficient between the level of oxidative stress and each parameter.

	r	p
2 CT-grade	0.573	0.001*
Sex	0.006	0.977
Age	0.227	0.245
BMI	–0.335	0.082
T-P	0.223	0.254
Alb	–0.399	0.035*
AST	0.170	0.387
ALT	–0.113	0.565
LDH	0.521	0.005*
ALP	0.384	0.043*
CRP	0.406	0.032*
WBC	0.302	0.118

2 CT-grade: two-step CT-grade (i.e., CT-grade which was divided into two groups: mild/moderate group and severe group.) **∗***p* < 0.05.

**Table 4:** Multiple regression analysis using oxidative stress as a dependent variable.

	Non-standardized coefficients					VIF
	β	SE	95%	C.I	p	
Constant	154.8	302.5	–472.5	782.3	0.61	
2 CT-grade	93	42.9	4	182	0.043*	1.67
Alb	–18.6	62	–147.4	110	0.76	3.18
LDH	1.18	0.75	–0.37	2.7	0.13	1.6
ALP	0.33	0.23	–0.16	0.82	0.17	1.22
CRP	–6.15	12	–31.1	18.8	0.61	3.62

SE: standard error, C.I: confidence interval, VIF: variance inflation factor ∗p < 0.05.

ANOVA: analysis of variance, p = 0.009, R = 0.69, R2 = 0.48.

## 4 Discussion

Patients with pMAC disease exhibited elevated levels of serum oxidative stress compared with those reported in healthy individuals. In addition, patients with severe disease – based on CT images – exhibited significantly higher levels of serum oxidative stress compared with those measured in the mild disease group. Numerous previous studies have associated lung diseases with oxidative stress. A previous study reported that serum catalase activity and the levels of lipid peroxides (i.e., 2-thiobarbituric acid reactive substances and 8-isoprostanes) – which are markers of oxidative stress in the serum of patients with bronchiectasis – were significantly increased. In contrast, the total antioxidant capacity and superoxide dismutase activity were decreased [7]. In IPF, which presents similar bronchodilation and structural changes in the lung to those observed with pMAC disease, it has been reported that the level of serum oxidative stress was increased with disease progression [10]. However, these studies did not identify a correlation between disease severity and early respiratory function (i.e., forced vital capacity, and diffusing capacity for carbon monoxide).

On the other hand, in a fundamental study performed by Akaki *et al.*, reactive oxygen intermediates (ROI) were produced by macrophages immediately after exposure to MAC and disappeared within 2 h [11]. However, it was concluded that ROI did not exert an antibacterial effect against MAC. In addition, a previous review investigated the association between *Mycobacterium tuberculosis* – a Mycobacterium similar to NTM – and oxidative stress. It was described that reactive oxygen species derived from immune cells, including host macrophages, were involved in the killing of *Mycobacterium tuberculosis* in the lungs of the host after infection. However, *Mycobacterium tuberculosis* produces bacterial superoxide dismutases and catalases, degrades superoxide and hydrogen peroxide, and is thought to be resistant to host oxidative stress. Therefore, it can be inferred that the bactericidal effect through ROI in NTM diseases may not be sufficient [12]. Based on these findings, it is suggested that the increase in the level of serum oxidative stress observed in this study reflected the ROI generated by inflammatory cells, including macrophages, as an immune response to infection with MAC. Tissue macrophages in the alveoli phagocytose MAC, and this process initiates the killing of MAC through ROI. However, MAC exhibits resistance against oxidative stress. Macrophages continue to produce ROI, and excessively produced ROI are released into the bloodstream.

The concentration of oxygen in the lung is high compared with those observed in other organs [13]. In addition, the lung is susceptible to oxidative stress. Therefore, it is likely that the lung may be exposed to excessive oxidative stress versus other organs. We compared the results of previously reported d-ROMs tests investigating other respiratory diseases with those obtained for pMAC disease in the present study. In chronic obstructive pulmonary disease, IPF, and sarcoidosis, the d-ROMs values were 267.8 ± 34.12 U.Carr (*p* < 0.001) [14,15], 383 ± 76 U.Carr (*p* < 0.001) [9], and 390 ± 25 U.Carr (*p* < 0.001), respectively [9].

The d-ROMs values obtained for pMAC disease in this study were significantly higher compared with those previously reported for other respiratory diseases. These results suggest that, in patients with pMAC disease, high oxidative stress is applied to the whole body. In addition, it is considered that further excessive production of inflammatory mediators from inflammatory cells may be promoted, leading to airway destruction.

Moreover, our findings suggested a correlation between the severity of imaging findings and markers of serum oxidative stress. According to Sheng-Wei Pan *et al.*, the CT findings of patients with pMAC disease who showed continuous discharge of the bacteria in sputum culture for >1 year were significantly more severe compared with those observed in patients who became MAC-negative [16]. Furthermore, Asakura *et al.* reported that the ratio of reduced permeability area to the whole lung area in a three-dimensional CT analysis was strongly correlated with the respiratory function and scores of St George's Respiratory Questionnaire. Of note, the investigators also identified a strong correlation between the cavity area and respiratory function [17]. These findings indicated a correlation between the severity of pMAC disease and imaging findings, as well as an indirect correlation between the level of serum oxidative stress and the severity of pMAC disease. However, currently, there are no quantitative markers available for assessing the severity of pMAC disease. Moreover, the results of lung function testing also depend on the cooperation of the individual and implementation methods. In addition, although CT is useful in the evaluation of the lesion area, it is not a quantitative index. Moreover, the facilities able to perform such evaluations are limited. Hence, measurement of serum oxidative stress may be useful as a simple indicator for assessing the severity of pMAC disease. Also, an evaluation of the role of the d-ROMs test as a quantitative index for the therapeutic effect on pMAC disease is warranted. This can be achieved by comparing the results of the d-ROMs test with the clinical course and imaging findings prior to and after the introduction of treatments.

This study was characterized by a few limitations. Firstly, this was a retrospective study involving a limited number of patients (n = 28). Thus, the influence of selection bias cannot be excluded. Secondly, it has been reported that the level of serum oxidative stress is increased in numerous diseases. Therefore, the potential effects of other non-NTM diseases cannot be excluded. Finally, this was a cross-sectional study, and the relationship between the level of serum oxidative stress and prognosis, or changes in the level of serum oxidative stress due to treatment, etc. are unknown. Prospective studies investigating the progression of pNTM, changes in the level of serum oxidative stress due to treatment, or association with prognosis, are required to establish the clinical significance of the level of serum oxidative stress in the diagnosis and treatment of pNTM.

## 5 Conclusions

The level of serum oxidative stress was elevated in patients with pMAC disease. Therefore, oxidative stress may be caused by a mechanism related to the infection of the lungs by MAC. Furthermore, the level of serum oxidative stress was higher in patients with severe disease versus that reported in patients with mild disease. Collectively, the findings of the present study suggest that oxidative stress may be involved in the process of airway destruction in patients with pMAC disease.

## Declarations

### Author contribution statement

Hiroki Wakabayashi: Conceived and designed the experiments; Analyzed and interpreted the data; Wrote the paper.

Yasuo Matsuzawa: Conceived and designed the experiments; Perfomed the experiments; Analyzed and interpreted the data.

Sho Hayakawa, Tamako Irie, Hagino Rikitake: Performed the experiments.

Ichiro Tatsuno: Analyzed and interpreted the data.

### Competing interest statement

The authors declare no conflict of interest.

### Funding statement

This research did not receive any specific grant from funding agencies in the public, commercial, or not-for-profit sectors.

### Additional information

No additional information is available for this paper.
